# Canine Skin and Conjunctival Swab Samples for the Detection and Quantification of *Leishmania infantum* DNA in an Endemic Urban Area in Brazil

**DOI:** 10.1371/journal.pntd.0001596

**Published:** 2012-04-10

**Authors:** Sidney de Almeida Ferreira, Rodrigo Souza Leite, Leonardo Trindade Ituassu, Gregório Guilherme Almeida, Daniel Menezes Souza, Ricardo Toshio Fujiwara, Antero Silva Ribeiro de Andrade, Maria Norma Melo

**Affiliations:** 1 Departamento de Parasitologia, Instituto de Ciências Biológicas, Universidade Federal de Minas Gerais, Belo Horizonte, Brasil; 2 Centro de Desenvolvimento da Tecnologia Nuclear, Comissão Nacional de Energia Nuclear, Campus da Universidade Federal de Minas Gerais, Belo Horizonte, Brasil; University of Pittsburgh, United States of America

## Abstract

**Background:**

We evaluated kDNA PCR/hybridization and quantitative real-time PCR (qPCR) targeting the gene of DNA polymerase of *Leishmania infantum* for CVL diagnosis and assessment of parasite load in clinical samples obtained invasively and non-invasively.

**Methodology/Principal Findings:**

Eighty naturally infected dogs from an endemic urban area in Brazil were used. Animals were divided into two groups based on the presence or absence of CVL clinical sings. Skin biopsies, bone marrow, blood and conjunctival swabs samples were collected and submitted to *L. infantum* DNA detection. In addition, anti-*Leishmania* antibody titers were measured by Immunofluorescence antibody test. The symptomatic dogs had increased titers compared to asymptomatic dogs (P = 0.025). The frequencies of positive results obtained by kDNA PCR/hybridization for asymptomatic and symptomatic dogs, respectively, were as follows: right conjunctiva, 77.5% and 95.0%; left conjunctiva, 75.0% and 87.5%; skin, 45.0% and 75.0%; bone marrow, 50.0% and 77.5%; and blood, 27.5% and 22.5%. In both groups, the parasite load in the skin samples was the highest (P<0.0001). The parasite loads in the conjunctival swab and bone marrow samples were statistically equivalent within each group. The parasite burden in conjunctival swabs was higher in the dogs with clinical signs than in asymptomatic dogs (P = 0.028). This same relationship was also observed in the bone marrow samples (P = 0.002). No differences in amastigotes load in the skin were detected between the groups.

**Conclusions:**

The conjunctival swab is a suitable clinical sample for qualitative molecular diagnosis of CVL. The highest parasite burdens were detected in skin regardless of the presence of VL-associated clinical signs. The qPCR results emphasized the role of dogs, particularly asymptomatic dogs, as reservoirs for CVL because of the high cutaneous parasite loads. These results may help to explain the maintenance of high transmission rates and numbers of CVL cases in endemic urban regions.

## Author Summary

Visceral leishmaniasis (VL) is considered the most lethal manifestation among the diseases caused by genus *Leishmania*. The main control measures for VL are: diagnosis and early treatment of human cases, insecticide vector control, and euthanasia of seropositive dogs. Although these strategies have been continuously applied in Brazil, the number of VL cases has increased and this disease still poses as a serious public health problem. Belo Horizonte is an endemic urban area in Brazil and it is considered by the Ministry of Health the third most affected Brazilian metropolitan region by VL. In the context of prophylaxis, the correct diagnosis of infected dogs is critical for the VL control because dogs represent the main domestic reservoir. However, the serological diagnosis techniques used in large-scale for canine screening in Brazil have important limitations including the difficulty of diagnosing asymptomatic dogs. Molecular methods based on Polymerase Chain Reaction (PCR) and real-time PCR (qPCR) have greatly improved VL diagnosis. Based on this, we aimed to evaluate different canine clinical samples for qualitative VL diagnosis by PCR and quantification of parasite load by qPCR in dogs from Belo Horizonte. The possible implications of results are discussed with emphasis on skin and conjunctival swab samples.

## Introduction

Visceral leishmaniasis (VL) is a severe zoonotic disease in Latin America that is caused by the protozoan *Leishmania infantum* [ = *L. chagasi*]. The parasite is transmitted through the bite of infected female phlebotomine sand flies. The prevalence of VL has been expanding throughout the world [1], and Brazil is one of six countries that account for 90% of all recorded human VL cases worldwide [2]. VL is the most severe clinical manifestation of *Leishmania* infection and it has high lethality in cases without suitable treatment [3].

In the context of VL prophylaxis, the rapid and accurate diagnosis of infected dogs is critical for the control of this zoonotic disease because they represent the main domestic reservoir [4]. Dogs have a close relationship with humans in both rural and urban areas, and canine cases usually precede human cases [4], [5]. Therefore, although VL remains more prevalent among dogs than humans, the presence of infected dogs may increase the risk for human infection in some situations [6].

The precise diagnosis of canine VL (CVL) is complex. The different available diagnostic techniques include parasitological demonstration, immunological tests and the detection of parasite DNA.

The VL control strategy based on the euthanasia of seropositive dogs in Brazil has been criticized predominately because the disease burden has not diminished over time [7], [8]. Although substantial technological improvements have been made, there is no gold standard technique or absolutely precise method for CVL diagnosis [9]. In most cases, a final diagnosis of CVL is obtained using a combination of distinct tests.

In Brazil, serological techniques based on the detection of anti-*Leishmania* antibodies, such as enzyme-linked immunosorbent assay (ELISA) and immunofluorescence antibody test (IFAT) are used for the large-scale screening of canine populations [10]. However, these methods have limitations related to their sensitivity and specificity that may lead to inconsistent results [11].

The detection of *Leishmania* DNA based on polymerase chain reaction (PCR) represents an alternative for VL diagnosis with highly sensitive, specific and versatile methods [12]. With these techniques, several types of canine clinical samples including bone marrow aspirates, spleen, lymph node, urine, blood, conjunctival swabs and skin biopsies can be used for VL diagnosis [13], [14], [15], [16]. PCR has been suggested as a useful method to detect subclinical infections and as a possible addition to serological tests to definitively diagnose inconclusive cases that exhibit low antibody titers or cross-reactivity [17]. In recent years, quantitative real-time PCR (qPCR) has demonstrated high sensitivity in detecting low parasitic loads [18]. Furthermore, qPCR is a reliable technique to diagnose CVL and monitor tissue parasite load in treated dogs in countries where the treatment is permitted [19], [20].

The present work was designed to evaluate canine clinical samples that were obtained invasively and non-invasively in two contexts. First, this study determined their usefulness for CVL molecular diagnosis by conventional PCR in naturally infected dogs with and without clinical signs compatible with this disease. Additionally, parasite burdens were assessed using qPCR. This work was conducted in Belo Horizonte, the capital of Minas Gerais, Brazil, where the CVL prevalence was estimated around 7.63% in more than 300,000 screened dogs [21], [22]. VL is urbanized in this city, which the Ministry of Healht considers one of Brazilian metropolitan region most affected by this disease [8], [23].

## Materials and Methods

### 1. Ethics Statement

Experiments with dogs were performed in compliance with the guidelines of the Institutional Animal Care and Committee on Ethics of Animal Experimentation (“Comitê de Ética em Experimentação Animal” – CETEA, national guidelines, Law number 11.794, 8/10/2008) from Universidade Federal de Minas Gerais (UFMG); protocol number 183/08 was approved on 04/03/2009.

### 2. Dogs

Eighty naturally infected mongrel dogs of both sexes and unknown ages destined to be euthanized were obtained from the Municipal Zoonotic Diseases Control Department of Belo Horizonte during routine surveillance. All animals had positive ELISA and IFAT and/or conventional parasitological test results. The dogs were submitted to a thorough physical exam and an aliquot of serum from each dog was obtained for biochemical evaluations. Serum proteins levels were assessed by the Biuret reagent; albumin and globulin concentrations were also estimated (BIOCLIN, Brazil). Serum urea was measured using a colorimetric enzymatic method (BIOCLIN, Brazil). In this case, sample absorbances at 510 nm were obtained using a spectrophotometer (Epoch, Biotek, USA). Serum creatinine was assessed using the colorimetric kinetics method (Cobas Mira Classic, Roche, Germany).

Based on the physical examination and the serum sample results, dogs were divided in two groups. Group 1 was composed of dogs without any suggestive clinical manifestations of CVL (n = 40). Group 2 was composed of dogs that presented clinical signs associated with VL, such as lymphadenopathy, alopecia, severe weight loss, onychogryphosis, keratoconjunctivitis, skin lesions, a low albumin/globulin ratio and/or high levels of creatinine and urea (n = 40). Ten healthy dogs that tested negative for CVL and were destined for adoption comprised the negative control group. This last group was kept in the kennel of Biological Sciences Institute of UFMG under controlled, isolated conditions with proper nutritional supply. Serum samples from all dogs examined in this study were resubmitted to ELISA and IFAT tests that were performed in the Serology Laboratory of the Parasitology Department of UFMG.

### 3. Clinical samples

Prior to sample collection, the dogs were anesthetized with 2% Xilazine (2.2 mg/kg, Syntec, Brazil) and 2.5% Thiopental (9.0 mg/kg, Cristália, Brazil). For DNA extraction, 1 mL of peripheral blood was collected from each dog in tubes containing Ethylenediamine tetraacetic acid (EDTA). The same volume of blood was collected in tubes without EDTA and centrifuged at 415 *g*; serum was collected for serodiagnosis and biochemical evaluation. Skin biopsies were obtained from the internal surface of the left ear using 5.0-mm sterile punches. Bone marrow aspirates were collected from the sternum using sterile needles. The injection site was previously cleaned with polyvinylpyrrolidone-iodine (Vansil, Brazil). Each aspirate was divided into two fractions. The first fraction (200 µL) was transferred to sterile tubes for DNA purification and the second was used for the parasitological test. All samples were kept on ice during transportation and stored at −20°C until use. Conjunctival swabs were obtained separately from both lower conjunctivas using sterile cotton swabs manufactured for bacteriological isolation. The swab tips were broken off, transferred to DNase-free tubes and stored at −20°C until use.

### 4. Parasitological test

The presence of parasites was investigated using a fraction of the bone marrow aspirates added to Novy-McNeal-Nicolle medium (NNN) with 12% rabbit's defibrinated blood overlaid with α-Minimum Essential Medium (GIBCO BRL, USA) containing 10% fetal calf serum (CULTILAB, Brazil), streptomycin (1.0 µL/mL) and penicillin (100 U/mL) (GIBCO BRL, Life Technologies, USA). The cultures were examined by optical microscopy and were subcultured three times over a 10-day period, after which all of the culture tubes were examined again. In addition, slide smears were prepared from skin biopsies and bone marrow samples. The slides were stained using the modified Giemsa method (Bioclin, Brazil) according to the manufacturer's protocol and subsequently observed under an optical microscope at 1000× magnification. These slides were analyzed when cultures were negative or exhibited signs of contamination.

### 5. Enzyme linked immunosorbent assay

Total serum IgG was measured using a previously described technique [24] with minor modifications. Briefly, the antigen used for the ELISA was a total soluble antigenic preparation from cultured promastigotes from *L. infantum*, strain MHOM/BR/1967/ BH46 that were disrupted by ultrasound (40 Hz) on ice and centrifuged at 150 *g* for 10 min. Individual wells of 96-well microplates were coated with the soluble antigen at a final concentration of 2 µg/mL in 0.05 M carbonate buffer (pH 9.6). A volume of 100 µL per well was used. Blocking was performed using PBS with 0.2% Tween-20 and 2% casein (Sigma, USA). To detect IgG, 100 µL of individual canine serum samples was serially diluted and transferred into each well. After proper treatment, 100 µL of peroxidase-conjugated rabbit anti-dog IgG (Sigma, USA) (diluted 1∶2000) was added to each well. Each well then received 100 µL of a mixture containing a 40% solution (w/v) of ortho-phenylenediamine in phosphate/citrate buffer (pH 5.0) and 30 volumes of H_2_O_2_. Absorbance values at 492 nm were measured in an automatic ELISA reader (Bio-Rad Model 550, USA). The cut-off values established for each reaction were the mean absorbance values +2 standard deviations from Brazilian dogs obtained from nonendemic areas for *L. infantum* [10].

### 6. Immunofluorescence antibody test

IFAT was performed according to an established method [25] using promastigotes from the *L. infantum*, strain described above. This strain is routinely used in Parasitology Department of UFMG for the detection of anti-*Leishmania* antibodies. The parasite cells used in this test were grown to stationary phase in liver infusion tryptose (LIT) medium [26] and the samples were considered positive when fluorescence was observed at dilutions ≥1∶40.

### 7. DNA Extraction

DNA was extracted from the blood and bone marrow samples using the GE Healthcare Illustra Blood Genomic Prep Mini Spin kit. Peripheral blood (1 mL) was added to 3.0 mL of red blood cell lysis solution [(10 mM KHCO_3_, 155 mM NH_4_Cl, and 0.1 mM EDTA (pH 8.0)]. The protocol was then performed following the manufacturer's recommendations. A total of 200 µL of bone marrow aspirate was processed in the same manner as the blood samples. However, treatment with the red blood cell lysis solution was omitted. The final volume of each DNA solution was 100 µL. Samples were then stored at 4°C until use.

DNA from the skin biopsies was purified using the Wizard SV Genomic DNA Purification System (Promega USA) following the manufacturer's protocol. The final volume of each DNA solution was 160 µL and the samples were stored at 4°C until use.

DNA from each conjunctival swab was extracted separately using the phenol-chloroform purification method. A mixture of lysis buffer solution [50 mM Tris, 50 mM NaCl, and 10 mM EDTA (pH 8.0)], 1% Triton X-100 and proteinase K (250 µg/mL) was added to each cotton swab. The mixture was incubated for 2 h at 56°C, eluted from the cotton swab, transferred to a PLG-H phase-lock gel tube (Eppendorf, Germany) and then mixed with 500 µL of 75% Tris-saturated phenol (Sigma) with 25% chloroform–isoamyl alcohol. The aqueous phase was separated from the organic phase by centrifugation at 12,000 *g* at 4°C for 5 min, and the supernatant was transferred to a new PLG-H phase-lock gel tube. DNA extraction was repeated twice, first with 500 µL of a 50% phenol and 50% chloroform–isoamyl alcohol solution and then with 100% chloroform–isoamyl alcohol. DNA was precipitated in one volume of isopropanol-sodium acetate followed by a 75% ethanol wash. The resulting DNA pellet was suspended in 30 µL of autoclaved ddH_2_O.

### 8. Conventional PCR

Conventional PCR was performed using primers specific to the *Leishmania* sp. kinetoplast DNA (kDNA) minicircle conserved region, as described elsewhere [27]. Here, 200 ng of each of the following primers were used: 5′-(G/C)(G/C)(C/G)CC(A/C)CTAT(A/T)TTACACAACCCC-3′ and 5′-GGGGAGGGGCGTTCTGCGAA- 3′ (GIBCO, São Paulo-Brazil). DNA samples at a final concentration of 1.0 ng/µL from *L. infantum* (MHOM/BR/1973/BH46 strain) and *L. braziliensis* (MHOM/1975/BR/M2903 strain) served as positive controls. DNA extracted from a dog with a confirmed infection was also used as a positive control. DNA from a healthy dog known to be negative for leishmaniasis served as a negative control. Furthermore, mixtures without DNA were included in all tests. From each naturally infected dog, 1.0 µL of DNA preparation from each clinical sample was submitted to the PCR. PCR amplifications were performed using a PTC-100 thermocycler (MJ Research, USA). All amplification products were analyzed on 2% agarose gels stained with ethidium bromide. The expected size of the amplified target was 120 base pairs (bp).

### 9. Hybridization

After the PCR assay, 10 µL of each amplification product was applied to a nylon membrane using a Bio Dot apparatus (Hybri-dot Manifold, BRL). Each well received 110 µL of 0.4 M NaOH and 25 mM EDTA (pH 8.0) denaturation solution; the membranes were then rinsed with 2× SSC (0.3 M NaCl and 0.3 mM sodium citrate) and dried. The DNA was fixed to the surface by UV crosslinking (0.12 J/cm^2^).

Cloned kDNA minicircles from *L. infantum* were labeled with ^32^P[α]dCTP using the random primer method (Invitrogen, USA). Hybridization conditions were reproduced as explained elsewhere [28]. Hybridization was also performed using cloned kDNA minicircle probes from *L. braziliensis* according to the same experimental design explained for *L. chagasi* probes.

### 10. Real-time PCR

The parasite loads were calculated by real-time PCR according to a method described elsewhere [29], [30] with minor modifications. Conjunctival swab, bone marrow and skin samples (240 DNA preparations) were evaluated. The parasite burdens were estimated using the following primers: Forward, 5′ TGT CGC TTG CAG ACC AGA TG 3′ and Reverse, 5′ GCA TCG CAG GTG TGA GCA C 3′. These primers amplified a 90 bp fragment of a single-copy-number *L. infantum* DNA polymerase gene (GenBank accession number AF009147). Canine housekeeping β-actin gene was used as endogenous control in order to normalize initial DNA concentrations and to verify sample integrity. The primers used to amplify a 307-bp fragment of β-actin were as follows: Forward, 5′ CTTCTACAACGAGCTGCGCG 3′ and Reverse, 5′ TCATGAGGTAGTCGGTCAGG. PCR was carried out in a final volume of 10 µL containing 0.7 pmol of each β-actin primer or 0.8 pmol of DNA polymerase primers, 2× SYBR GREEN reaction master mix (Applied Biosystems, USA), 4.0 µl of DNA with a final concentration of approximately 20 ng/µL and an enough volume of ultrapure water. Reactions were processed and analyzed in an ABI Prism 7500 Sequence Detection System (SDS Applied Biosystems, Foster City, CA, USA). The followed steps were programmed: an initial denaturation at 95°C for 10 min followed by 40 cycles of denaturation at 95°C for 15 s and annealing/extension at 60°C for 1 min. Standard curves were prepared using known amounts of TOPO PCR 2.1 plasmids (Invitrogen, USA) that contained canine genes of β-Actin or *L. infantum* DNA polymerase gene. The respective cycle threshold (C_T_) values obtained for samples were calculated based on the corresponding standard curve; the results were defined as the number of parasites per canine cells. Overall, 20% of samples were chosen randomly and resubmitted to PCR to verify technique reproducibility.

### 11. Statistical analysis

The frequencies of positive results obtained from all the clinical samples were compared separately within each group using the Pearson χ^2^ test with a 5% significance level. This same statistical tool was used to compare the results between groups.

Kappa index was used to assess the reproducibility of molecular tests. Sensitivity and specificity for PCR-hybridization performed with different clinical samples were calculated using 2×2 tables and the conventional parasitological test was adopted as a reference test. For the data with non-parametric distribution, the Mann-Whitney test was used for comparisons between two data sets. The Kruskal-Wallis test followed by Dunn's test was performed for the comparison of three samples simultaneously. In cases where data were distributed parametrically, Student's t test or one way ANOVA was used to compare two or three data sets respectively. The difference between the results was considered statistically significant at a *P* value<0.05. All calculations were performed using the Winpepi (PEPI for Windows) and GraphPad Prism 5.0 software programs.

## Results

First, the healthy dogs, which did not show any clinical signs of CVL, were submitted to the parasitological, serological and molecular tests described above. All of these procedures showed negative results for the control group. Additionally, all of the positive hybridization results obtained from the naturally infected dogs examined in this study were identified as *L. infantum* (data not shown). All negative and positive controls of assays carried out in this work showed expected results and validated the obtained data.

### 1. Serology

**Figure 1:**
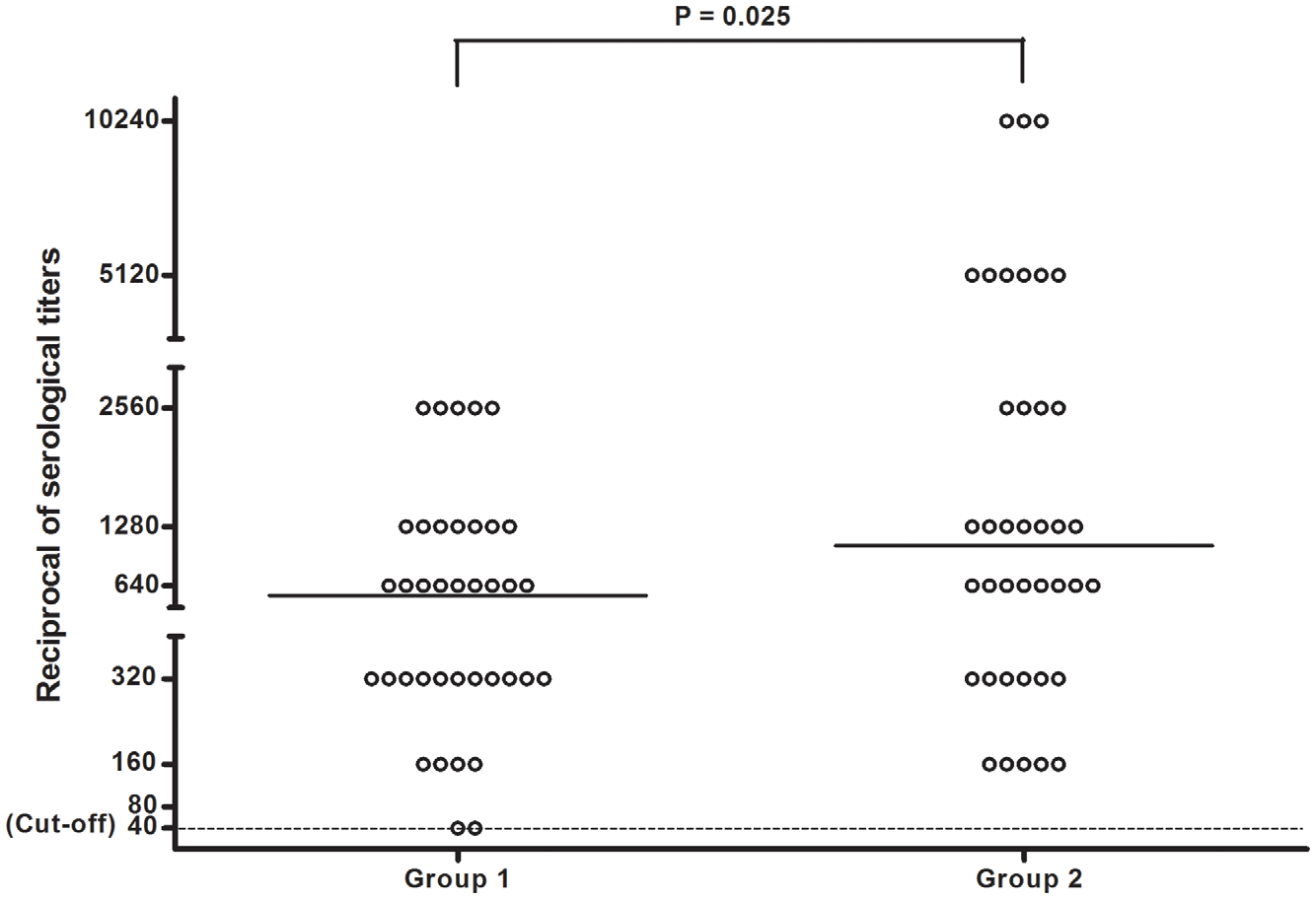
Reciprocal of serological titers obtained by IFAT for 80 naturally infected dogs. Group 1: 40 dogs with no clinical signs of CVL; Group 2: 40 dogs with clinical manifestations of CVL. The horizontal line in each titer distribution represents the geometric mean. Cut-off = 1∶40.

Three animals that did not exhibit any clinical signs of CVL had negative ELISA results. Two of these dogs were also negative by IFAT. Only one dog with clinical signs had a negative IFAT result. These animals had positive parasitological test results, confirming the presence of infection. All other dogs tested positive for CVL by both serological assays. The absorbance values obtained by ELISA were not statistically different between the groups (*P*>0.05) (data not shown). In the IFAT assays, the dilutions varied from 1∶40 to 1∶2,560 in the dogs from group 1 and from 1∶160 to 1∶10,240 in the dogs from group 2. Examination of the IFAT assay titers between groups revealed that the dogs with clinical signs of VL had higher titers than dogs without clinical signs (P = 0.025) (Figure 1).

### 2. Dogs without clinical signs: conventional PCR/hybridization and qPCR

The frequency at which conventional PCR/hybridization produced a positive result from the conjunctival swabs was calculated separately for the right and left conjunctiva in each group. Then, the positive detection rates in the different clinical samples were compared within each group. Approximately 20% of all DNA preparations were retested by conventional PCR and the same proportion of samples was retested using qPCR. The repetitions received different codes and were performed blinded. The agreement and kappa values were, respectively, as follows: PCR-hybridization, 93% and 0.85; real-time PCR, 100% and 1.0 (Table S1). The sensitivity and specificity measures are shown in table S2.

In group 1, the PCR/hybridization positive result rates for the clinical samples were as follows: right conjunctiva, 77.5% (31/40); left conjunctiva, 75.0% (30/40); skin, 45.0% (18/40); bone marrow, 50.0% (20/40); and blood, 27.5% (11/40) (Table 1). In this group, the positive results obtained from either the right or the left conjunctiva were statistically greater than those from any of the other clinical samples tested (*P*<0.05) (Table 1).

**Table 1:** Comparison between paired clinical samples according to the results obtained by PCR/hybridization test in group 1.

	*Positivity*	
*Positivity*	Right conjunctiva31/40 (77,5%)	Left conjunctiva30/40 (75%)
Skin18/40 (45%)	P = 0.003	P = 0.006
Bone marrow20/40 (50%)	P = 0.011	P = 0.021
Blood11/40 (27,5%)	P<0.001	P<0.001

The statistical significance shown for each comparison is based on the χ^2^ test.

**Figure 2:**
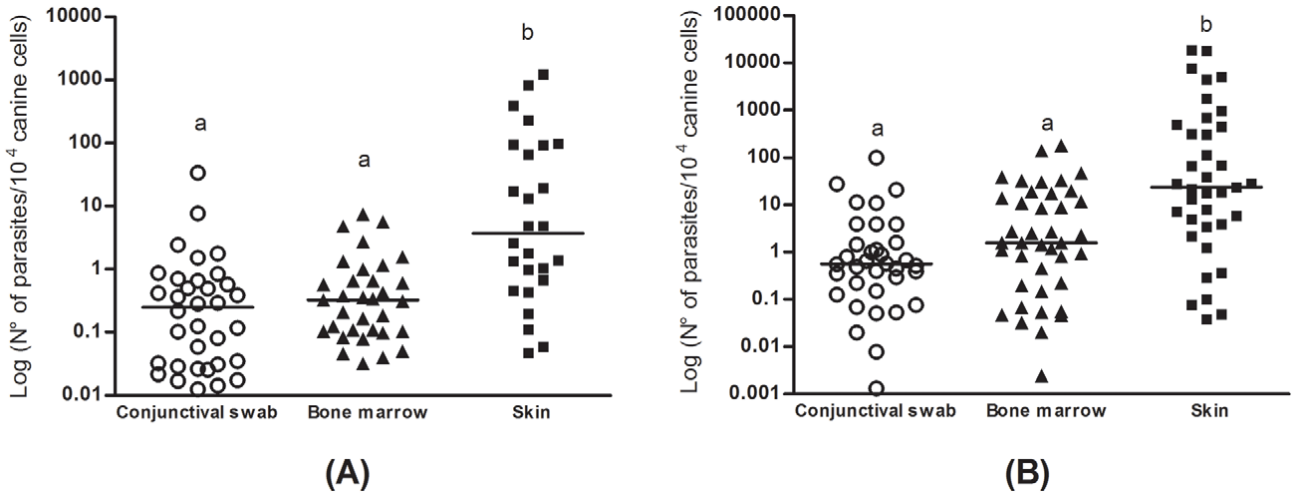
Parasite load in clinical samples of naturally infected dogs. (A): asymptomatic dogs; (B): dogs with clinical manifestations suggestive of VL. (a): No statistical difference among the measures indicated. (b) The parasite load in the skin was higher than those estimated in conjunctival swab and bone marrow samples (P<0.0001).

In the qPCR assays, the efficiency of standard curves was 97% and 100% for DNA polymerase and β-actin respectively. In group 1, the parasite burdens in the conjunctival swab and bone marrow samples were statistically equivalent (P>0.05). In contrast, the parasite load in the skin was higher than in the other clinical samples (P<0.0001) (Figure 2A).

### 3. Dogs with clinical signs: conventional PCR/hybridization and qPCR

In group 2, the positive result rates for PCR/hybridization were as follows: right conjunctiva, 95.0% (38/40); left conjunctiva, 87.5% (35/40); skin, 75.0% (30/40); bone marrow, 77.5% (31/40); and blood, 22.5% (9/40) (Table 2). The percentage of positive results in right conjunctival swabs was statistically higher compared to the other samples tested (*P*<0.05). On the other hand, there was no difference between the results obtained from the left conjunctiva and those from the skin or bone marrow (*P*>0.05). However, there was a statistical difference between the left conjunctiva and blood (*P*<0.001) (Table 2).

**Table 2:** Comparison between paired clinical samples according to the results obtained by PCR/hybridization test in group 2.

	*Positivity*	
*Positivity*	Right conjunctiva38/40 (95%)	Left conjunctiva35/40 (87.5%)
Skin30/40 (75%)	P = 0.012	P>0.05
Bone marrow31/40 (77.5%)	P = 0.023	P>0.05
Blood9/40 (22.5%)	P<0.001	P<0.001

The statistical significance shown for each comparison is based on the χ^2^ test.

The parasite loads in the conjunctival swabs and bone marrow were not statistically different (P>0.05). However, the parasite burden in the skin was statistically higher than those obtained from other clinical samples (P<0.0001) (Figure 2B).

### 4. Comparison between groups – molecular techniques

**Figure 3:**
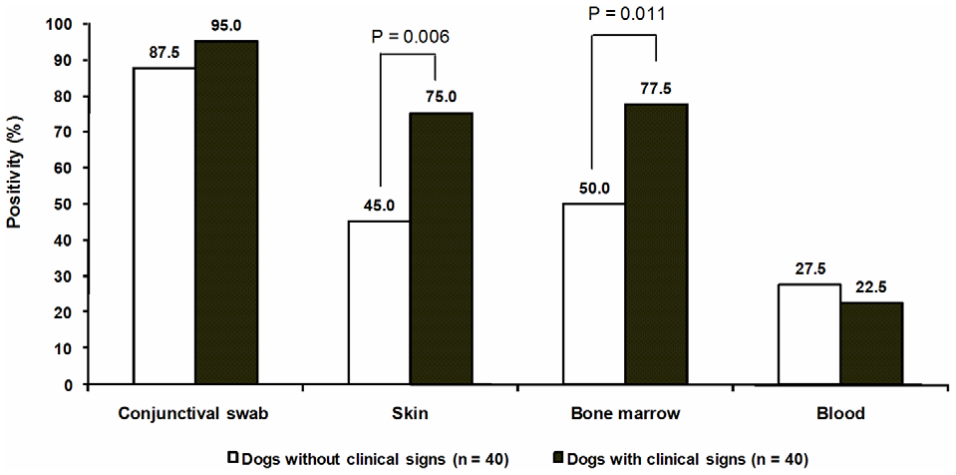
Positivity rates of conventional PCR followed by hybridization in 80 naturally infected dogs. The numbers above the bars indicate the percentage of positive dogs for all clinical samples. For conjunctival swab positivity rates, animals that tested positive in at least one of the two conjunctivas were considered positive.

There were no differences in the kDNA PCR/hybridization results when the right and left conjunctival samples were compared within and between groups (*P*>0.05) (data not shown). Therefore, the dogs were counted as positive in one of the conjunctivas or both. The rates of positive results in dogs exhibiting no clinical signs and those with clinical status were 87.5% (35/40) and 95.0% (38/40), respectively. These results were not statistically different (*P*>0.05) (Figure 3). The results from the other clinical samples were also compared between groups. The positive rates in the skin samples from dogs with clinical signs were statistically higher than from dogs without clinical signs (*P* = 0.006). Similarly, the positive rate in bone marrow samples was higher in the symptomatic group (group 2) compared to the group 1 (*P* = 0.011). In contrast, the positive results detected in the blood were not different between the two groups (*P*>0.05) (Figure 3).

**Figure 4:**
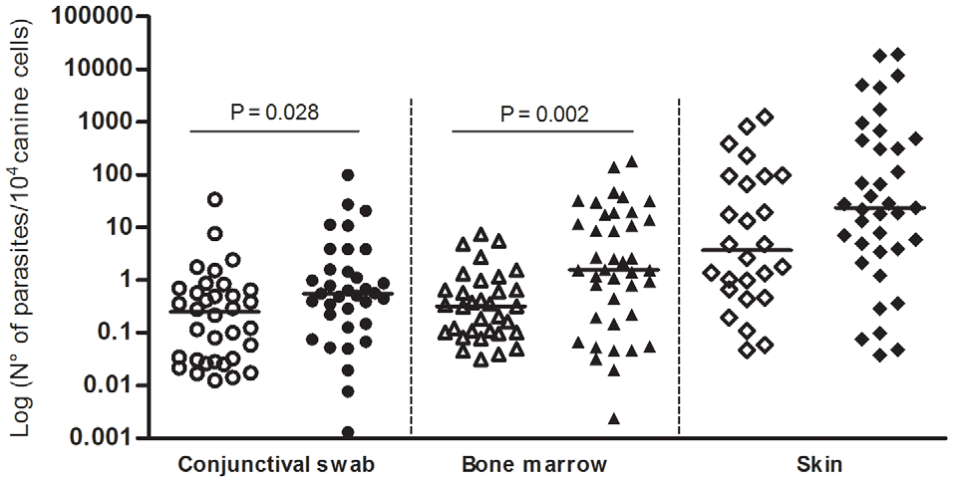
Comparison of parasite burdens in conjunctival swab, bone marrow and skin samples. White: naturally infected dogs without clinical signs; Black: naturally infected dogs with clinical signs. Comparisons were made in pairs considering the same kind of clinical sample.

Based on qPCR results, the parasite load from conjunctival swab in group 2 was higher than in group 1 (P = 0.028). The same relationship was also found for bone marrow (P = 0.002). However, no differences were observed in skin parasite load between groups (P>0.05) (Figure 4).

## Discussion

In Brazil, the main VL control measures that have been adopted are the diagnosis and early treatment of human cases, insecticide vector control, and euthanasia of seropositive dogs. Despite these efforts, the number of VL cases has increased in urban areas from Brazil, and VL remains a serious public health problem [31], [32]. In this context, infected dogs have been implicated in all registered VL outbreaks and epidemics. This study was performed in Belo Horizonte, a large Brazilian city where VL is prevalent [33]. Identification and selection of infected dogs were based on a combination of serological and parasitological tests owing to the diagnostic limitations of these techniques when they are used in isolation.

The combined use of ELISA and IFAT kits (Biomanguinhos/FIOCRUZ/MS) is recommended for a more efficient detection of CVL and the cut-off dilution adopted by the Ministry of Health for the IFAT is 1∶40. However, these serological tests have important limitations for routine use, and the combination of different methods is usually necessary for a precise diagnosis. The serological methods used in large-scale canine surveys may show false positive results due to cross-reactivity with other diseases, such as trypanosomiasis or cutaneous leishmaniasis (CL) [34], [35]. In the metropolitan region of Belo Horizonte, CL caused by *L. braziliensis* has previously been recorded in a small number of dogs [36]. Furthermore, these diagnostic methods may underestimate the actual prevalence of CVL in endemic regions [37], [38].

Although the conventional parasitological tests for VL have high specificity, they exhibit low sensitivity and also require invasive sample collection methods. Moreover, these techniques are time-consuming, are susceptible to microbiological contamination and are not suitable for the large-scale screening of canine populations [11]. On the other hand, molecular-based diagnostic techniques have become an important resource to improve CVL diagnosis because of its high sensitivity and specificity.

The molecular analyses of this work were divided in two parts. First, qualitative conventional PCR followed by hybridization was used to identify the proportion of dogs that exhibited *Leishmania* DNA in different clinical samples. In this case, the frequency of positive results obtained from skin samples was higher in dogs with clinical manifestations compared to asymptomatic dogs. This same relationship was also observed for bone marrow samples. Different CVL studies have demonstrated high positive indices for conventional PCR test of skin samples from dogs with different clinical signs, and it has been suggested that this technique based on use of ear skin could be the best procedure for CVL diagnosis [39]. However, the collection of ear skin samples is painful, bloody and invasive and requires local anesthesia and aseptic manipulation.

Bone marrow is often included in research on VL diagnosis and represents an important source of *L. donovani* complex parasites for parasitological tests because of the lymphoid tropism of this group of protozoans. PCR performed using DNA from the bone marrow has shown good sensitivity for CVL diagnosis [14], [16] indicating that this clinical sample is a suitable source of DNA for molecular diagnosis. However, the collection of bone marrow aspirates is invasive and traumatic for the dog. Complete anesthesia of the animal is necessary, and this usually results in opposition by dog owners. These limitations make this procedure unsuitable for large-scale surveys. Moreover, in the present study, the rates of positive results obtained from the bone marrow and skin using the kDNA PCR/hybridization method were lower than those from conjunctival swab, which involve a non-invasive collection method.

Although blood is a less invasive collection method, it had the lowest positive detection rate in both groups. The evaluation of blood for the diagnosis of CVL by PCR is still controversial. Different studies have reported that blood samples worked well for PCR-based CVL diagnosis [40], [41]. In contrast, other authors encountered problems with the use of blood samples for diagnosis, such as the presence of PCR inhibitors, variations in parasite load, and low sensitivity [42], [43]. At the present work, the smaller frequencies of positive results were obtained from blood. Almost all amplifications products from this clinical sample presented a lot of unspecific bands at agarose gel analysis. Probably, this decreased the specificity of the primers and might have reduced the sensitivity of hybridization. The occurrence of unspecific amplifications was particularly evident in this kind of sample and the real reasons of that and the dynamics of parasite load in canine blood remains unclear [42].

For qualitative molecular diagnosis, the conjunctival swab samples showed the best results with high percentages of positive results for both groups of dogs. In this experimental context, the frequency of positive results detected from the conjunctival swab samples did not appear to depend on the clinical status of the infected dogs. Although the IFAT has showed different results between dogs with and without clinical signs, the high positive detection rates obtained from conjunctival swab samples by kDNA PCR/hybridization were not dependent on anti-*Leishmania* antibody titers. In a longitudinal study on the detection of canine *Leishmania* infections by PCR, conjunctival swabs showed a slow positive conversion prior to seroconversion in areas with elevated CVL prevalence [44]. The ocular region can be reached by *Leishmania* through hematogenous dissemination [45] or by direct infection through phlebotomine bites around the dogs' eyes particularly in endemic regions where the vector density can be high [46]. Similar to a previous study, the frequencies of positive results in combined conjunctivas samples were greater than those in the separated conjunctivas [47]. However, the results between eyes were not statistically different. Thus, the use of just one ocular swab would be useful for large-scale qualitative PCR-based screening of dog populations.

In a second context, the molecular analyses were carried out to estimate the parasite burdens by qPCR in conjunctival swab, bone marrow and skin samples. All of these samples were submitted to qPCR performed under the same conditions. The conjunctival swabs were chosen because of their practicability, and bone marrow was tested because it usually contains a high number of amastigotes in infected dogs [16], [48]. Finally, skin was included because of the epidemiologically importance of this sample as the principal access point for the phlebotomines and as the primary site of initial infection in dogs.

Dogs that exhibited clinical signs of CVL exhibited increased parasite loads in conjunctival swabs and bone marrow compared to asymptomatic dogs. However, the parasite burdens of these samples were lower than in skin within each group. Our results suggest that parasitic density in the tissue of inner eyelids is low. Few *Leishmania* cells in conjunctival swabs were detected in another study, even when swabs from both eyes were combined [49]. It is probable that amastigotes reach and remain in the conjunctival epithelial tissue through the infiltration of a small number of infected macrophages [43]. Similar positive PCR results were found between lymph node and conjunctival swab samples indicating this specimen as an attractive alternative diagnostic sample [50]. According to our data, the number of *Leishmania* cells estimated for conjunctival swabs was equivalent to that for bone marrow in both groups of naturally infected dogs. Therefore, despite the known lymphoid tropism of *Leishmania*, the bone marrow samples did not exhibit the high diagnostic performance reported in previous studies [14], [51]. This result highlights the potential of a sample obtained non-invasively as an auxiliary resource for CVL molecular diagnosis.

Surprisingly, the parasite burdens in the skin were the highest in both groups of dogs. In addition, no differences were observed in skin parasite load between the two groups. In this case, the different antibody titers detected in the two groups of dogs did not appear to be correlated with parasite burden in the skin. The serological response correlated positively with the clinical status of dogs, confirming results found in other studies [52]. Manna et al (2006) demonstrated that infected asymptomatic dogs had higher parasite load in the skin than in lymph node. Additionally, the cutaneous parasitism remained high for 6 months [53]. When compared to parasite load estimated for bone marrow samples based on the Leishman Donovan Units technique, an increased parasite burden in the skin was also found [52]. Our results corroborate these findings in an endemic urban region in Brazil and emphasize the role of infected dogs, especially the asymptomatic dogs, as a reservoir. These animals represent a risk for sand fly infection because of the intense parasitism of their skin. As a matter of fact, the serological tests performed for the large-scale screening of dogs in Brazilian cities can fail to detect subclinical infections [17]. Additionally, a delay between the discovery of seropositive dogs and culling is common [7]. These factors associated with considerable skin parasitism of dogs, especially those without clinical signs, may contribute to the continual high transmission rates and prevalence of CVL cases in Belo Horizonte and possibly in other endemic regions of Brazil and world.

The molecular techniques used in this study showed a good reproducibility with high degree of agreement. These results indicate that these tests are reliable for the purposes described in this work.

Taken together, our results indicate that conjunctival swabs are suitable for qualitative molecular CVL diagnosis and that their widespread use should be considered. Nonetheless, these samples exhibited low parasite load in infected dogs sampled in this study, similar to the bone marrow samples. In spite of this, it was possible to accurately quantify *Leishmania* cells in dogs studied. This clinical sample associated with these molecular techniques could be useful for human VL diagnosis and must be investigated. The highest parasite burdens were detected in skin samples from both symptomatic and asymptomatic dogs. These results represent a great challenge for CVL control in VL endemic areas in Brazil, such as Belo Horizonte. This work can be seen as a basis for large-scale field studies to verify the epidemiological implications of the molecular techniques and canine clinical samples used here.

## Supporting Information

Table S1 **Reproducibility of molecular methods.**
(DOC)

Table S2 **Sensitivity and specificity of PCR-hybridization performed with different clinical samples for canine visceral leishmaniasis diagnosis.**
(DOC)
