# Supplementary material for: Canine Skin and Conjunctival Swab Samples for the Detection and Quantification of Leishmania infantum DNA in an Endemic Urban Area in Brazil
Source: PLoS Negl Trop Dis. 2012 Apr 10;6(4):e1596. doi: 10.1371/journal.pntd.0001596 (PMC3323509; doi:10.1371/journal.pntd.0001596)
Supplement: Table S1 — Reproducibility of molecular methods. (DOC) [file pntd.0001596.s001.doc]

**Supplementary table 1**

Reproducibility of molecular methods.

| ***Molecular techniques*** | ***Agreement (%)*** | ***Kappa (95% CI)*** |
| --- | --- | --- |
| cPCR-hybridizaton | 93 | 0.85 (0.73-0.98) |
| qPCR | 100 | 1.00 (1.00-1.00) |

CI: confidence interval;

cPCR: conventional PCR;

qPCR: real-time PCR;

Approximately 20% of DNA preparations from both groups of naturally infected dogs were retested and submitted to reproducibility test.
