# Supplementary material for: Canine Skin and Conjunctival Swab Samples for the Detection and Quantification of Leishmania infantum DNA in an Endemic Urban Area in Brazil
Source: PLoS Negl Trop Dis. 2012 Apr 10;6(4):e1596. doi: 10.1371/journal.pntd.0001596 (PMC3323509; doi:10.1371/journal.pntd.0001596)
Supplement: Table S2 — Sensitivity and specificity of PCR-hybridization performed with different clinical samples for canine visceral leishmaniasis diagnosis. (DOC) [file pntd.0001596.s002.doc]

**Supplementary table 2**

Sensitivity and specificity of PCR-hybridization performed with different clinical samples for canine visceral leishmaniasis diagnosis.

| *Clinical samples* | *N = 50** | *Sensitivity %***  *(95% CI)* | *Specificity %***  *(95% CI)* |
| --- | --- | --- | --- |
| Conjunctival swab*** | Group 1 | 86 (70.6-93.7) | 67 (41.7-84.8) |
|  |  |  |  |
| Bone marrow | Group 1 | 54 (38.2-69.5) | 93 (70.2-98.8) |
|  |  |  |  |
| Skin | Group 1 | 51 (35.6-67) | 100 (79.6-100) |
|  |  |  |  |
| Blood | Group 1 | 31 (18.6-48) | 100 (79.6-100) |
|  |  |  |  |
| Conjunctival swab*** | Group 2 | 97 (84.3-99.4) | 61 (38.6-79.7) |
|  |  |  |  |
| Bone marrow | Group 2 | 84 (68.2-93.1) | 78 (54.8-91) |
|  |  |  |  |
| Skin | Group 2 | 78 (61.2-89) | 72 (49.1-87.5) |
|  |  |  |  |
| Blood | Group 2 | 25 (13.2-42) | 94 (74.2-99) |

*The 10 negative dogs were added to both groups of 40 infected dogs;

**Sensitivity and specificity were calculated based on parasitological culture test as gold standard;

***Positive result was considered for at least one of the conjunctivas;

CI: confidence interval;

Group 1: naturally infected dogs without clinical manifestations;

Group 2: naturally infected dogs with clinical manifestations.
